# Scoping Review: The Role of Tocotrienol-Rich Fraction as a Potent Neuroprotective Agent

**DOI:** 10.3390/ijms26167691

**Published:** 2025-08-08

**Authors:** Elvira Yunita, Muhammad Luqman Nasaruddin, Nur Zuliani Ramli, Mohamad Fairuz Yahaya, Hanafi Ahmad Damanhuri

**Affiliations:** 1 Department of Biochemistry, Faculty of Medicine, Universiti Kebangsaan Malaysia Medical Centre (UKM-MC), Kuala Lumpur 56000, Malaysia; elvirayunita@unib.ac.id (E.Y.); hanafi.damanhuri@ppukm.ukm.edu.my (H.A.D.); 2 Department of Biochemistry and Molecular Biology, Faculty of Medicine and Health Sciences, University of Bengkulu, Bengkulu 38371, Indonesia; 3 Department of Anatomy, Faculty of Medicine, Universiti Teknologi MARA, Sungai Buloh 47000, Selangor, Malaysia; zulianiramli@uitm.edu.my; 4 Department of Anatomy, Faculty of Medicine, Universiti Kebangsaan Malaysia Medical Centre (UKM-MC), Kuala Lumpur 56000, Malaysia; mfairuzy@ukm.edu.my

**Keywords:** antioxidant, brain, tocotrienol-rich fraction, neuroprotection

## Abstract

Tocotrienol-rich fraction (TRF), a subtype of vitamin E, has recently been reported to demonstrate promising neuroprotective properties. However, it remains to be fully determined how it confers protection in the brain. This scoping review aimed to explore and understand the intricate role of TRF in promoting and preserving neuronal well-being. A systematic literature search, based on the framework by Arksey and O’Malley and adhering to the PRISMA-ScR guidelines, was conducted across several databases, including PubMed, Scopus, and Web of Science (WOS), using the following phrases and Boolean operators: (“tocotrienol-rich fraction”) AND ((“neuroprotect”) OR (“cognit”) OR (“brain”)). The search yielded a total of 24 eligible articles, shortlisted based on predetermined inclusion and exclusion criteria established at the outset of the study. The findings highlight a diverse array of TRF-related studies, both in vivo and in vitro, that revealed functional mechanisms through which TRF confers neuroprotection. These include, but are not limited to, antioxidant and anti-inflammatory effects via attenuation of superoxide dismutase (SOD) activity and pro-inflammatory mediators; regulation of metabolic pathways; regulation of neuronal genes, proteins, and maintenance of cellular functions; and subsequent improvements in memory and cognitive performance in animal models following TRF treatment. The convergence of these neuroprotective effects suggests that TRF holds potential as a supplement to support healthy ageing or, at the very least, slow neurodegeneration by mitigating pathological changes that often begin insidiously before the onset of symptoms associated with cognitive decline.

## 1. Introduction

TRF is a compound derived from palm oil that typically contains 25% tocopherol and 75% tocotrienol (both of which are subclasses of vitamin E) or other variations in which tocotrienol is the dominant component [1,2,3,4,5]. To a certain extent, it exhibits favourable antioxidant properties compared to tocopherols, owing to its unique molecular structure, which enhances its ability to penetrate membrane tissues effectively, particularly in fat-rich organs such as the brain and liver [2,6].

Previous findings have suggested several ameliorative effects of TRF across various parameters in both preclinical and clinical settings. Notably, TRF supplementation in patients with hypercholesterolemia led to significant reductions in cardiovascular and metabolic markers, with additional evidence of positive carryover effects observed in some of the studied patients [7,8,9]. In one study, a synergistic effect with lovastatin was observed, resulting in improvements in the high-density lipoprotein to low-density lipoprotein (HDL/LDL) ratio [9]. The same observation was also seen in diabetic patients, where despite showing no significant hypoglycaemic changes, cholesterol and cardiovascular markers decreased significantly after 60 days of TRF treatment in these patients [10]. It is postulated that TRF’s ability to ameliorate atherosclerotic progression is due to its capacity to modulate peroxisome proliferator-activated receptors (PPARs), as demonstrated in mouse studies and reviewed by Ramanathan et al. [11]. Furthermore, TRF has also been found to prevent muscle ageing. One study demonstrated that TRF promotes the renewal and regeneration of skeletal muscle by enhancing energy and amino acid synthesis [12]. Likewise, TRF modulated the oxidative status in myoblasts to improve replicative senescence, a process contributing to sarcopenia observed during ageing [13]. It is also interesting to note that TRF supplementation reduces oxidative stress and subsequently improves spatial learning and memory in aged rats compared to healthy controls [14]. Cumulatively, TRF is suggested to be a viable option as a supplement to improve overall well-being, as it has shown significant improvements in inflammation and lipid peroxidation status in both healthy individuals and those with underlying conditions, as recorded in a recent systematic review involving more than 2000 patients across 30 studies [15].

It is interesting to note that TRF also confers protection in neurodegenerative conditions. In studies on Alzheimer’s disease (AD), tocotrienol analogues were shown to improve Aβ oligomerization and disaggregation of preformed fibrils in vitro [16]. TRF supplementation was also found to alter overall protein expression related to metabolic pathways and oxidative phosphorylation in the brains of APP transgenic mice compared to healthy controls [17]. Moreover, in a different study, brain metabolite profiling of the transgenic model showed modest improvement in perturbed metabolic pathways along with enhanced memory function following TRF supplementation [18]. Similar observations were reported in the same AD model, where TRF supplementation attenuated Aβ plaque deposition in the brain, ultimately leading to improved cognitive function [19]. Furthermore, in a separate proteomic study, when compared to levodopa, a commonly prescribed medication for Parkinson’s disease, TRF showed overlapping protein expression patterns [20]. Both TRF and levodopa were found to potentially modulate key cellular processes in neuroblastoma cells, providing promising avenues for neuroprotection.

Currently, despite numerous studies that have suggested the plausible effects of TRF on various health parameters, the extent to which its neuroprotective mechanisms support or maintain neuronal health remains inadequately explored. This review is therefore conducted to understand the intricate role of TRF in promoting and preserving neuronal well-being by examining findings from various experimental models and, where available, clinical studies. It also aims to provide an updated overview of the mechanisms involved in neuroprotection. It is also hoped that this effort will support the potential of TRF as a viable, natural alternative intervention to protect against or reduce the damage associated with ageing, whether general, accelerated, or pathological as seen in conditions such as AD, PD, and/or other forms of neurodegeneration. Given that such neurodegenerative changes may begin before the onset of cognitive symptoms, and, considering that current pharmacological treatments are largely providing symptomatic relief rather than curative, the need for such an alternative is deeply warranted.

## 2. Results

A total of 114 papers were retrieved from three databases: 24 from PubMed, 42 from Scopus, and 48 from Web of Science (WOS). In total, 55 articles were excluded due to duplication, while 8 were deemed irrelevant as they were not primary research articles. Two papers could not be retrieved due to access limitations. Additionally, 24 papers were excluded from the scoping review because they focused solely on the individual effects of tocopherol or tocotrienol, addressed only metabolic illnesses, or were unrelated to the effects of TRF on the brain. Only 24 articles met the inclusion criteria set at the onset of the review. The details of these articles are summarized in Table 1, where the eligible studies are categorized based on the potential effects of TRF in the following domains: (1) antioxidant and anti-inflammatory actions, (2) gene and protein expression, (3) neuronal memory, cognitive processes, and cellular regeneration, and (4) maintenance of cellular and organelle morphology. Findings from the Risk of Bias (ROB) analysis of the eligible animal studies indicated a low risk of bias in areas such as random housing of animals, baseline similarity, random selection for outcome assessment, and avoidance of other potential sources of bias (see Supplementary Materials S1).

### Ameliorative Effects of TRF on Brain Health: Insights from In Vitro and In Vivo Studies

TRF was shown to regulate the activity of immune-responsive cells. In one study, TRF-treated astrocytes exposed to toxic levels of glutamate showed reduced lipid peroxidation and increased cell viability [21]. The same study reported enhanced antioxidant defences, including elevated glutathione (GSH) levels, reduced malondialdehyde (MDA), and fewer apoptotic astrocytes [21]. Similarly, TRF treatment improved the viability of lipopolysaccharide (LPS)-treated macrophages and reduced their activation, thereby dampening the release of pro-inflammatory markers [22]. In another study, primary cell lines differentiated into a neural lineage and subjected to glutamate-induced excitotoxicity showed signs of recovery, as evidenced by the reduced levels of reactive oxygen species (ROS) observed in TRF-treated cells [23]. Likewise, neuronal cell survival was further improved in a separate study, where TRF treatment decreased ROS levels and suppressed glutamate receptor and neuron-specific enolase (NSE) expression, therefore attenuating neuronal stress and injury [24].

Notably, long-term TRF supplementation in healthy aged rats restored vitamin E concentrations and raised the levels of superoxide dismutase (SOD) and glutathione peroxidase (GSH-Px), allowing for sustained oxidative protection [25]. Additionally, in the hippocampus of middle-aged healthy rats, TRF was found to restore complex I activity, thereby preventing mitochondrial dysfunction as the rats aged [26]. Moreover, in a separate study, TRF supplementation restored age-associated alterations in arginine metabolites, particularly in the entorhinal cortex and cerebellum of aged rats, which correlated with improved memory functions [27].

This overall general protective effect was also observed in oxidative stress-related animal models. In one study, TRF pretreatment in a fenitrothion-induced oxidative stress model preserved cerebellar tissue morphology, reduced malondialdehyde (MDA) content, and increased SOD activity compared to controls [28]. Moreover, in a separate study, rats exposed to toxic levels of lead to induce oxidative stress showed reduced hippocampal lead accumulation and increased erythrocyte SOD activity, which collectively improved histopathological changes following TRF treatment [29]. Likewise, TRF-treated rats of vascular dementia model showed reversed oxidative parameters and prevention of memory loss [30]. The same study also reported increased expression of platelet-derived growth factor-C (PDGF-C) in the hippocampus, suggesting improved neuronal health [30].

In addition, it was also demonstrated that DNA damage was significantly reduced and antioxidant activity was positively modulated in a TRF-treated AD transgenic mouse model relative to age-matched controls [31]. In relation to this, a lower number of cell deaths were reported in TRF-treated SH-SY5Y neuroblastoma-treated cells as compared to amyloid-beta treated cells [32]. Interestingly, in a different study, TRF-treated neuroblastoma cells showed similar improvements in antioxidative and AD-related GSK3B and TAU protein markers (of tau hyperphosphorylation and neurofibrillary tangle formation) compared to controls [20]. The study also noted additive neuroprotective effects in the form of upregulation of the GLUT 4 gene when TRF combined with Vitamin D [20].

**Table 1:** Data extracted from eligible papers.

No	Paper	Types of Studies	Model	Effects of TRF
1	[21]	In vitro	Astrocytes induced by glutamate	TRF treatment ↑ cell viability and GSH levels compared to the untreated group. Additionally, TRF pre-treatment ↓ MDA levels compared to the control and tocopherol groups.
2	[23]	In vitro	Oxidative stress in human neuroblastoma SH-SY5Y cells induced by 6-hydroxydopamine (6-OHDA)	TRF pre-treatment in 6-OHDA-induced neuroblastoma cells ↑ cell viability, ↓ ROS generation and CAT activity, ↑ SOD activity, and ↑ dopamine receptor D2 gene expression compared to the untreated groups (treated with 6-OHDA without TRF supplementation).
3	[22]	In vitro	RAW264.7 macrophages culture	TRF treatment ↓ IL-6 levels, NO generation, prostaglandin E2 release, and cyclooxygenase-2 gene expression in LPS-stimulated macrophages when compared to the control group, which consisted LPS-stimulated macrophages that did not receive TRF supplementation.
4	[24]	In vitro	The transgenic mouse ES cell line, 46C	TRF significantly ↓ ROS levels, as well as the expression of glutamate receptor N-methyl-D-aspartate-1 (NMDA-1), Kainate-1, and neurone-specific enolase in the transgenic cell line compared to the untreated group (ethanol + cells).
5	[33]	In vivo	T2DM/vascular dementia in rats	TRF supplementation in diabetic rats ↓ blood insulin, fasting blood glucose, brain GSH, and SOD levels compared to the T2DM group. TRF dose-dependent therapy ↓ structural abnormalities of hippocampal histopathology in T2DM-induced vascular dementia (VaD) rats, while preserving the compactness of the stratum pyramidale and preventing neuronal cell loss.
6	[25]	In vivo	Healthy rats	Administration of TRF ↑ vitamin E levels, SOD activity, GSH-Px activity, and ↓ protein carbonyl levels (a biomarker of oxidative stress) in the brain compared to the untreated group. Supplementation with TRF did not cause any changes in the modulation of CAT activity in the brain.
7	[28]	In vivo	Fenitrothion-treated rats	Pre-treatment with TRF ↓ MDA levels, SOD activity, and ↑ GSH levels compared to the fenitrothion group. TRF pre-treatment also did not result in a significant increase in acetylcholinesterase activity in the brains of rats treated with fenitrothion compared to the untreated group.
8	[31]	In vivo	Transgenic mice	Supplementation with TRF ↓ SOD activity, but did not significantly affect in GSH-Px activity when compared to the untreated group. The non-supplemented mice showed ↑ in DNA damage.
9	[20]	In vitro	Human SH-SY5Y neuroblastoma cells	TRF supplementation affected 81 differentially expressed proteins (53 ↑, 28 ↓), while levodopa modulated 57 proteins (32 ↑, 25 ↑). A core set of 32 proteins were commonly regulated by both TRF and levodopa.
10	[34]	In vitro	Neuroblastoma cell line, SK-N-SH	TRF supplementation ↑ p-AKT, ↓ glycogen synthase kinase-3β (GSK3β), ROS, and tubuline-associated unit (TAU) expression, but did not affect the expression levels of insulin signalling proteins when compared to the untreated cells.
11	[33]	In vivo	Vascular dementia/aluminum chloride-induced rats	Administration of TRF ↑ memory performance (elevated plus maze test) and PDGF-C protein levels compared to the untreated ones, suggesting improved neuronal health. TRF supplementation ↓ plasma myeloperoxidase, and brain-thiobarbituric acid reactive substance levels compared to the untreated group (aluminum chloride-induced rats without TRF supplementation).
12	[27]	In vivo	Young and old rats	TRF administration ↓ levels of L-ornithine, glutamine, glutamate/GABA ratio, and L-citrulline/L-arginine ratio, primarily in the entorhinal cortex and cerebellum of the elderly group compared to the untreated group. These reductions led to ↓ NO production, which may decrease neurotoxicity. TRF also ↑ the cognitive performance in aged rats, as measured by OFT and MWM, when compared to the younger group.
13	[35]	In vivo	Alzheimer’s disease mice model	Treatment with TRF ↓ gene expression in the mRNA processing, epidermal growth factor receptor (EGFR1), p53, the PI3K-Akt-mTOR, NF-kB, T cell receptor, TNF-alpha, and mitogen-activated protein kinase (MAPK) signalling pathways in the APPswe/PS1dE9 transgenic AD mice model group compared to the untreated ones.
14	[17]	In vivo	Alzheimer’s disease/APPswe/PS1dE9 double transgenic mice	TRF treatment ↑ overlapping expression of APP and PTPRA in the hippocampus of transgenic mice than in the untreated group. TRF treatment also altered the expression of metabolic system proteins related to AD, PD, Huntington’s disease, and oxidative phosphorylation in APPswe/PS1dE9 double transgenic mice compared to the untreated group.
15	[36]	In Vitro	Mice hippocampal HT22 neuronal cell line	TRF treatment ↑ cell proliferation at the S phase (undifferentiated stage) and activated the BDNF/TrkB signalling pathway in a time-dependent manner in the HT22-neuronal cell line compared to the untreated cells. It ↑ memory through neural plasticity by ↑ the phosphorylation of AMPA receptor component GluA1, promoting LTP.
16	[19]	In vitro and In vivo studies	Human neuroblastoma cell line SH-SY5Y; double transgenic Alzheimer’s disease mice (AβPP/PS1)	TRF interfered with the aggregation of Aβ42 in human neuroblastoma cells without reducing cell viability. Compared to the untreated group, AβPP/PS1 mice ↑ cognitive performance following TRF supplementation. TRF treatment ↓ amyloid beta deposition in the brains of APP/PS1 mice. However, TRF had no significant effect on microglial activation.
17	[37]	In vivo	Healthy/F0 (female) and F1 (male) rats	TRF supplementation ↑ the levels of α-tocotrienol and cognitive abilities (MWM test) compared to the untreated ones. Supplementing the mothers of the animal model with TRF ↑ cognitive function and behavioural performance in offspring, as assessed by the MWM test.
18	[38]	In vivo	Multiple Sclerosis rat model	TRF-treated rats with multiple sclerosis ↑ the overall distance travelled, although there were no alterations observed in exploratory behaviour during the OFT, including the average duration of time spent in the centre, as compared to the untreated group. The TRF group preserved callosum morphology, ↓ MDA levels in the isocortex compared to the control group (no cuprizone and TRF).
19	[14]	In vivo	Young and old rats	TRF-treated aged rats ↑ exploratory behaviour, memory, spatial learning, SOD, CAT, GSH-Px levels and ↓ DNA damage, ↓ MDA levels compared to untreated rats.
20	[18]	In vivo	AβPP/PS1 double transgenic mice	TRF supplementation ↑ rearing duration, indicating enhanced exploration in transgenic mice compared to the untreated mice using OFT. TRF treatment ↑ spatial learning, memory, acetylcholine, L-aspartic acid, L-tyrosine, and L-glutamic acid levels in transgenic mice compared to the untreated group.
21	[32]	In vitro	SH-SY5Y neuroblastoma cell culture treated with Aß	TRF-treated neuroblastoma cells supplemented with Aß peptide ↓ DNA damage, the number of apoptotic cells, and ↑ cell survival compared to untreated ones (Aß peptide-induced without TRF supplementation).
22	[26]	In vivo	Healthy/male young and adult rats	Middle-aged rats showed ↓ hippocampal mitochondrial mass integrity compared to the younger rats. The results indicated that age had an impact on mitochondrial function. TRF supplementation ↑ mitochondrial complex I activity in the hippocampus of adult rats compared to PKO-treated rats.
23	[39]	In vivo	Stress-induced rats/Sprague Dawley rats	The plasma corticosterone levels, the rates of cell division and the survival of granule cells in the dentate gyrus were not affected by the addition of TRF 200 mg/kg BB for 21 days compared to the untreated group.
24	[29]	In vivo	Lead (Pb) poisoning on rats	Supplementation with TRF in rats with lead poisoning ↓ the occurrence of apoptotic-like features in the cornusammonis subregion of the hippocampus compared to the control group. The concentration of total TRF in the brain ↑ in rats with lead poisoning than in the untreated group (rats with lead poisoning but without TRF supplementation). In addition, supplementation with TRF in lead-poisoned rats also ↑ SOD activity and had no effect on MDA levels compared to the untreated group.

↑ and ↓ refers to either (increased or upregulated (↑) and decreased or downregulated (↓)). Abbreviations: 6-OHDA: 6-hydroxydopamine; AMPA: α-amino-3-hydroxy-5-methyl-4-isoxazolepropionic acid; APP: amyloid β/A4 precursor protein; BDNF-TrkB: brain-derived neurotrophic factor/Tropomyosin receptor kinase-B; CAT: catalase; GSH: glutathione; GSH-Px: glutathione peroxidase; IL-6: interleukin-6; LPS: lipopolysaccharide; MDA: malondialdehyde; MWM: Morris water maze; NO: nitric oxide; OFT: open-field test; PDGF-C: platelet-derived growth factor C; PKO: palm kernel oil; PTPRA: receptor-type tyrosine-protein phosphatase alpha; LTP: long-term potentiation; SOD: superoxide dismutase; T2DM: Type 2 Diabetes Mellitus.

Moreover, it is important to note that TRF exerts effects at both the transcriptomic and proteomic levels. In a study involving TRF supplementation in a double-transgenic mouse model of AD, several pathways were found to be modulated and/or improved in the hippocampus as a result of the treatment. The transcriptomes involved were found to implicate pathways such as (1) cell survival and stress signalling (including oxidative stress), (2) gene and protein regulation, (3) inflammation and immune response pathways, and (4) genes involved in the progression of neurodegenerative diseases, among which are AD, PD, and p53 and Wnt signalling pathways [35]. Furthermore, proteomic analysis of different brain regions of TRF-treated AD mice revealed significant alterations in the proteins involved in several overlapping functions, including metabolic (energy-related) processes, antioxidant defence, and overall proteomic signatures typically associated with AD pathology, particularly amyloid beta accumulation [17]. Lastly, a pathway enrichment analysis conducted after the proteomic analysis of TRF- and/or levodopa-treated neuroblastoma cells revealed differentially expressed proteins associated with pathways related to PD, AD, amyotrophic lateral sclerosis (ALS), Huntington’s disease (HD), and other pathways of neurodegeneration [20].

It is interesting to note that the molecular-level changes observed in cells and tissues following TRF treatment in animals are consistent with behavioural data from these studies, which show improved cognitive and locomotor functions in both aged and AD models [14,18,19,36], as well as studies involving developmental (pre- and post-natal TRF-treated offspring) and demyelination models [37,38].

**Figure 1:**
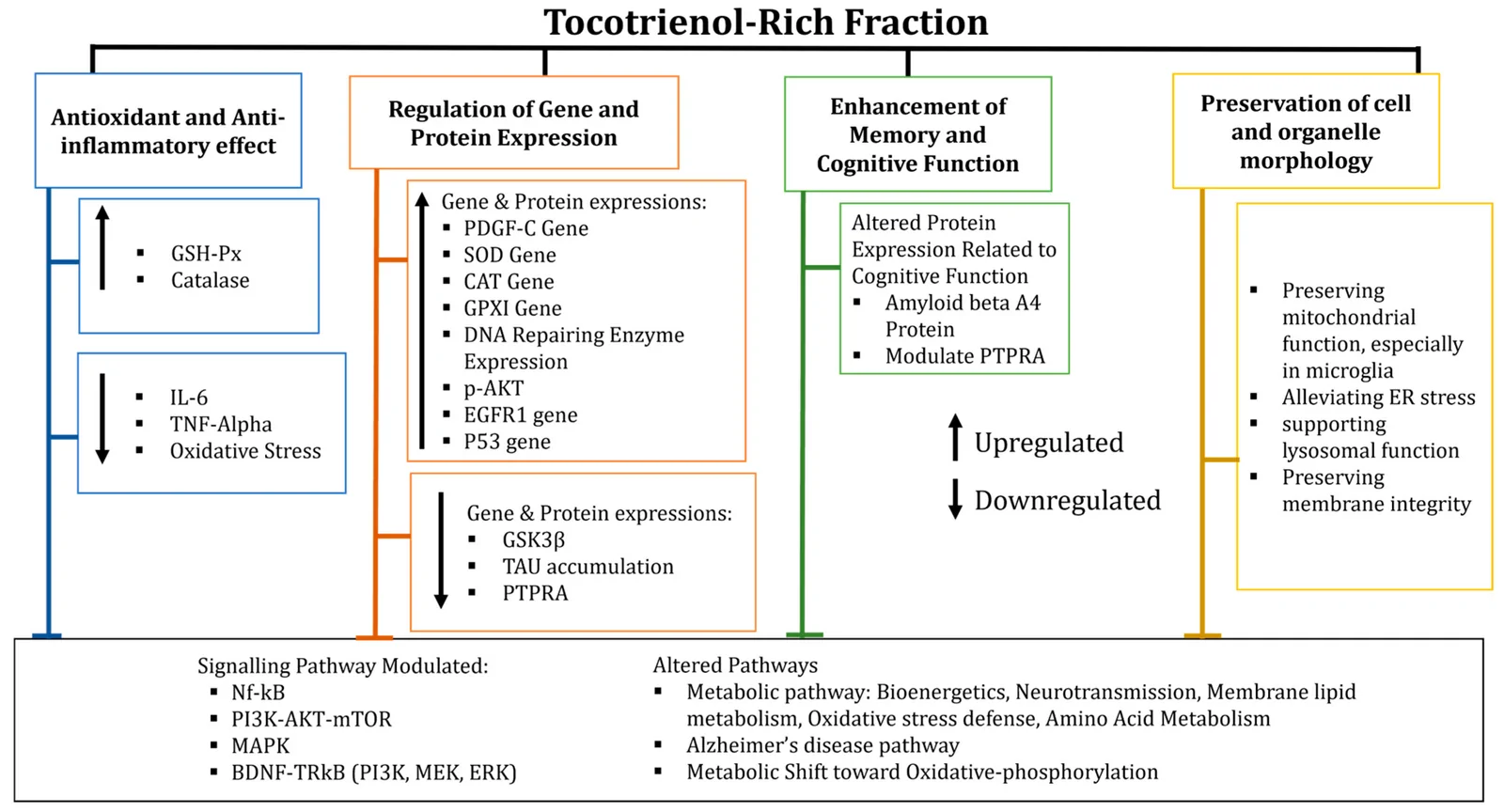
The proposed role of TRF-mediated neuroprotection. Abbreviations: BDNF-TRkB: brain-derived neurotrophic factor/Tropomyosin receptor kinase-B; CAT: catalase; EGFR: epidermal growth factor receptor-1; ER: endoplasmic reticulum; GPX1: glutathione peroxidase 1; GSH-Px: glutathione peroxidase; GSK3β: glycogen synthase kinase-3 beta; IL-6: interleukin-6; MAPK: Mitogen-Activated Protein Kinase; p-AKT: phosphorylated Akt; PDGF-C: platelet-derived growth factor-C; PI3K-AKT-mTOR: phosphatidylinositol-3-kinase (PI3K)/AKT/mechanistic target of rapamycin; PTPRA: protein tyrosine phosphatase receptor type-A; SOD: superoxide dismutase; TNF: tumour necrosis factor.

Collectively, these findings support the proposed role of TRF-mediated neuroprotection. TRF can modulate antioxidant and anti-inflammatory responses, regulate gene and protein expressions, maintain the functions of organelles, and improve cognitive function (Figure 1).

## 3. Discussion

The findings of our scoping review reveal several interconnected roles of TRF in promoting and/or preserving neuronal well-being. In both healthy ageing and chronic neurodegeneration, TRF’s effects, as demonstrated across various in vitro and in vivo studies, have been consistently linked predominantly with antioxidant properties. Multiple pathways modulated by TRF were proposed in the review, including, but not limited to, metabolic and mitochondrial bioenergetic processes, antioxidant defence mechanisms, inflammatory and immune response pathways, and regulatory networks associated with neurodegeneration. This diverse range of mechanisms through which TRF exerts its neuroprotective effects across different experimental models appears to converge on a unifying theme: regulation of mitochondrial function and its potential modulation of microglial activity.

Among the various hallmarks of ageing, mitochondrial dysfunction remains central to its progression [40,41,42]. It is considered to be one of the main mechanisms contributing to elevated ROS levels found in ageing [43,44]. ROS are by-products of mitochondrial respiration, primarily generated at Complexes I and III of the electron transport chain (ETC). Redox balance is characteristically restored by neutralizing ROS through enzymatic and non-enzymatic means involving the GSH–GSH-Px–GSSG cycle, SOD, and catalase. Cellular oxidative damage occurs when redox homeostasis fails and ROS production exceeds the cell’s antioxidant capacity.

Moreover, unmitigated oxidative stress often initiates a vicious cycle in which ROS damage mitochondrial DNA (mtDNA), further exacerbating mitochondrial dysfunction [45,46]. A causal relationship between age-related mitochondrial dysfunction and inflammation has been highlighted in a recent study, whereby leaked mtDNA in the cytosol of microglia activates the cGAS-STING pathway [47] that primarily drives interferon-1 (IFN-1)-mediated inflammation and age-associated neurodegeneration [48]. The study showed a loss of neuronal density in the hippocampus, accompanied by cognitive impairment in the aged mouse model [48]. Building on this, promotion of microglial ageing through functional decline of mitochondria in microglia could also heighten the generation of ROS and inflammatory mediators [49,50,51]. Studies have also demonstrated that leaked mtDNA, ROS, or ATP can trigger downstream activation of the NLRP3 inflammasome, a multiprotein complex that activates key pro-inflammatory pathways that leads to the production of pro-inflammatory cytokines [52,53,54,55,56].

Furthermore, it is important to note that the maintenance of neuroinflammation and neuronal homeostasis is also regulated by microglial autophagy. Impairment of mitophagy, a specialized form of autophagy targeting damaged mitochondria, causes the accumulation of defective mitochondria, leading to sustained ROS buildup that progressively worsens inflammation and cellular dysfunction [57,58,59,60]. In a typical ageing setting, it is reported that a significant decline in mitophagy activity in both the prefrontal cortex and the dentate gyrus of the hippocampus was recorded in older mice relative to young [61]. However, in aberrant neurodegenerative conditions such as AD, an elevated number of damaged mitochondria has been reported, with nearly a 60% reduction in microglial mitophagy activity observed in the hippocampus of AD mice compared to wild-type controls. [62]. This impairment is thought to be due to amyloid beta itself and the accumulation of the amyloid precursor protein-c terminal fragmentation (APP-CTF) in early AD models and human post-mortem sporadic AD brains that showcase clear morphological mitochondrial changes [63,64]. It is important to note that removal of stressed/damaged mitochondria, as seen in AD pathophysiology, is *Parkin*-dependent mitophagy [65]. Likewise, in PD patients, a robust inflammatory response including IL-6, was observed following PRKN/PINK1, which promoted mtDNA release and mitochondrial damage [66]. Overexpression of Parkin in AD-mouse model showed improvement of mitophagy and a reduction in amyloid-beta levels [67].

It is vital to note that compromised mitochondrial function not only affects microglial regulation and activity, but also influences neuronal health [68]. This highlights the interconnectedness of these systems and the need for interventions that target one or all of them simultaneously. Suppression of NLRP3 inflammasome activation by promoting mitophagy, as observed in previous studies [69,70], could theoretically reduce ROS levels, mitochondrial damage-associated molecular pattern (DAMP) release, and cell apoptosis. At least in one study we reviewed, mitochondrial dysfunction in the hippocampus of rats was found to begin in midlife, and TRF supplementation was shown to restore mitochondrial Complex I activity [26], supporting its potential protective role against neurodegenerative conditions associated with Complex I deficiency [71]. Whether this improvement also contributes to reduced inflammation or improved neuronal status remains unclear, and represents a current limitation where the literature on the topic appears lacking. However, supplementation has, in some cases in the hippocampal region, demonstrated benefits that may suggest a potential link worth further exploration. A recent study found TRF to exhibit no effect on stress-induced dentate gyrus cell loss and corticosterone levels [39]. However, TRF treatment in the entorhinal cortex exhibited a reversal of age-associated changes in arginine metabolites [27]. Furthermore at the molecular and cellular levels studies have demonstrated histopathological improvements in the hippocampus [29,30], increased total vitamin E levels in the brain [25], and lowered oxidative marker levels [23,28]. These changes collectively help explain the observed enhancement in memory, cognitive function, and locomotor activity in several animal models following treatment [14,30,38] whilst preserving neuronal survival and function.

Interestingly two studies we reviewed highlighted the amelioration of ROS in glutamate-induced oxidative stress in neural cells and astrocytes [3,24]. Moreover, we also found that TRF directly affects immune cells both in LPS-activated human monocytes and RAW264.7 macrophages by promoting the release of anti-inflammatory mediators and attenuating NF-κB regulation and subsequent suppression of COX-2 [22,72]. Interestingly, the suppression of the NF-κB is translated in model of diabetes and caused alleviated cognitive functions [73]. There is no study in the literature that investigated the direct effects of TRF on microglia. To date, there is only a single study of δ-tocotrienol (a subset of TRF) on microglial cells that exhibited downregulation of IL-1B and reduced transcription of COX-2 [74]. The range of evidence suggests that TRF may indeed modulate the NF-κB pathway.

In addition, several studies reviewed here showcase TRF’s potential to improve AD conditions or modulating protein expressions in PD [17,18,19,20,31,32,34,35,36]. Even though the proposed mechanisms involved remain speculative, they seem to centre on several key pathways. Notably, TRF restores mitochondrial energy homeostasis and enzyme activity while also modulating broader metabolic processes, as well as influencing amyloid-beta processing. Taken together, TRF supplementation in AD mouse models was associated with attenuation of amyloid-beta deposition and overall improvements in memory and cognitive functions. Whether these effects are primarily driven by restored mitochondrial function, increased mitophagy that clears damaged mitochondria, or microglial polarization towards the anti-inflammatory M2 phenotype (as reported in other antioxidant type [75,76]) with improved phagocytic capacity, the precise mechanism by which TRF exerts its antioxidant effects is not fully understood. It is plausible that these events occur concurrently, suggesting the pleiotropic effects of TRF in modulating ageing in general or specifically neurodegenerative conditions. Moreover, it also remains to be determined whether TRF acts upstream by directly and or indirectly modulating inflammasome, the cGAS–STING pathway, or the PINK1/Parkin pathway. This remains unknown and warrants for further investigation.

### Limitation of the Review and Future Directions

Most of the studies that were included are either in vivo or in vitro studies. There were no human clinical trials looking into the effects of TRF in the brain. The lack of clinical data restricts the generalizability of the findings to human populations. This scoping review also reported various formulations and dosages across experimental designs which potentially could compound the biological effects of TRF, the outcomes of which may have contributed to differences in the reported findings.

Furthermore, research related to AD has focused mostly on transgenic models instead of sporadic ones. Transgenic AD models is typically utilized to investigate early-onset familial AD, and do not capture the complexity of the sporadic AD that accounts for the majority of AD cases. Only one study from our review that utilized an Al-Cl_3_-rat model to mimic sporadic AD [33]. Future research should, in the long run, consider the use of sporadic models of AD to closely mimic disease pathogenesis and explore the potential use of TRF to improve AD conditions. In addition, as the studies reported here examine only a single time point, a longitudinal multi-omics study assessing various timepoints throughout the treatment period should be conducted to better understand the range of affected pathways. By doing so, we may be able to identify and optimally target critical time windows that offer the greatest potential for delaying disease progression or improving outcomes.

Moreover, TRF supplementation was found to be given after pathological onset. Given the number of promising findings from studies reviewed here, researchers should revert their focus to prevention rather than treatment. One study looking into the pre- and postnatal effects of TRF in rats showed notable cognitive performance with increased levels of α-tocotrienol in both plasma and brain [37]. In addition, a 19% decrease in clinical progression per year was observed in a mild-to-moderate AD group following α-tocopherol treatment [77], and consumption of diet-derived vitamin E demonstrated an association with decreased risk of developing AD in individuals without APOE ε4 [78].

It is also worth noting that the ‘gut–brain’ axis could potentially play a role in the pathogenesis of ageing and/or neurodegenerative diseases. A recent study has demonstrated the existence of a distinct microbial consortium that is associated with AD subjects, as compared to healthy controls and those with amnestic mild cognitive impairment [79]. Like many other antioxidants, TRF has the potential to positively modulate microbiomes to release metabolites that could exert neuroprotection and delay ageing or related degenerations [80].

## 4. Materials and Methods

This scoping review was conducted using Arksey and O’Malley’s methodological framework [81]. The methodology followed five main steps: (1) identifying the research question; (2) searching for the relevant studies and literature; (3) selecting the studies; (4) organizing and charting the data; and (5) summarizing the key findings and reporting the results [81]. Additionally, we adhered to the PRISMA Extension for Scoping Reviews (PRISMA-ScR) guidelines [58,82], which provide a standardized checklist for reporting scoping reviews. The complete PRISMA-ScR checklist is available in Supplementary Material S2. A risk of bias (RoB) assessment was also conducted for all animal studies included in the review using SYRCLE’s Risk of Bias (RoB) tool, with detailed results presented in Supplementary Material S1. A protocol was submitted into the Open ScienceFramework (Registered DOI: https://doi.org/10.17605/OSF.IO/TD2YW).

### 4.1. Identifying the Research Question

Tocotrienol-rich fraction (TRF) has significant potential as a neuroprotective agent, based on current studies. The main research question this scoping review addressed, as defined by its objectives and scope, was “How does tocotrienol-rich fraction (TRF) contribute to the promotion and preservation of neuronal well-being, and what are the underlying mechanisms of its neuroprotective effects as demonstrated across experimental and clinical studies?”

### 4.2. Searching the Relevant Studies and the Literature

This review incorporates research findings sourced from the Scopus, PubMed, and Web of Science (WOS) databases. The final search was conducted on 29 April 2025. The search strategy focused on the following phrases and Boolean operators: (“tocotrienol-rich fraction”) AND ((“neuroprotect*”) OR (“cognit*”) OR (“brain”)). Only studies that met the following criteria were included: (1) primary research articles; (2) related to the effects of TRF on the brain; and (3) full-text availability. Studies were excluded if they focused solely on tocopherol (another sub-family of vitamin E), exclusively on individual tocotrienol analogues without referring to TRF, or if they investigated the effects of TRF in combination with other substances without isolating the specific effect of TRF itself. Research examining TRF’s biological activity in organs other than the brain was also excluded, as it was not relevant to the scope of this review.

### 4.3. Study Selection

**Figure 2:**
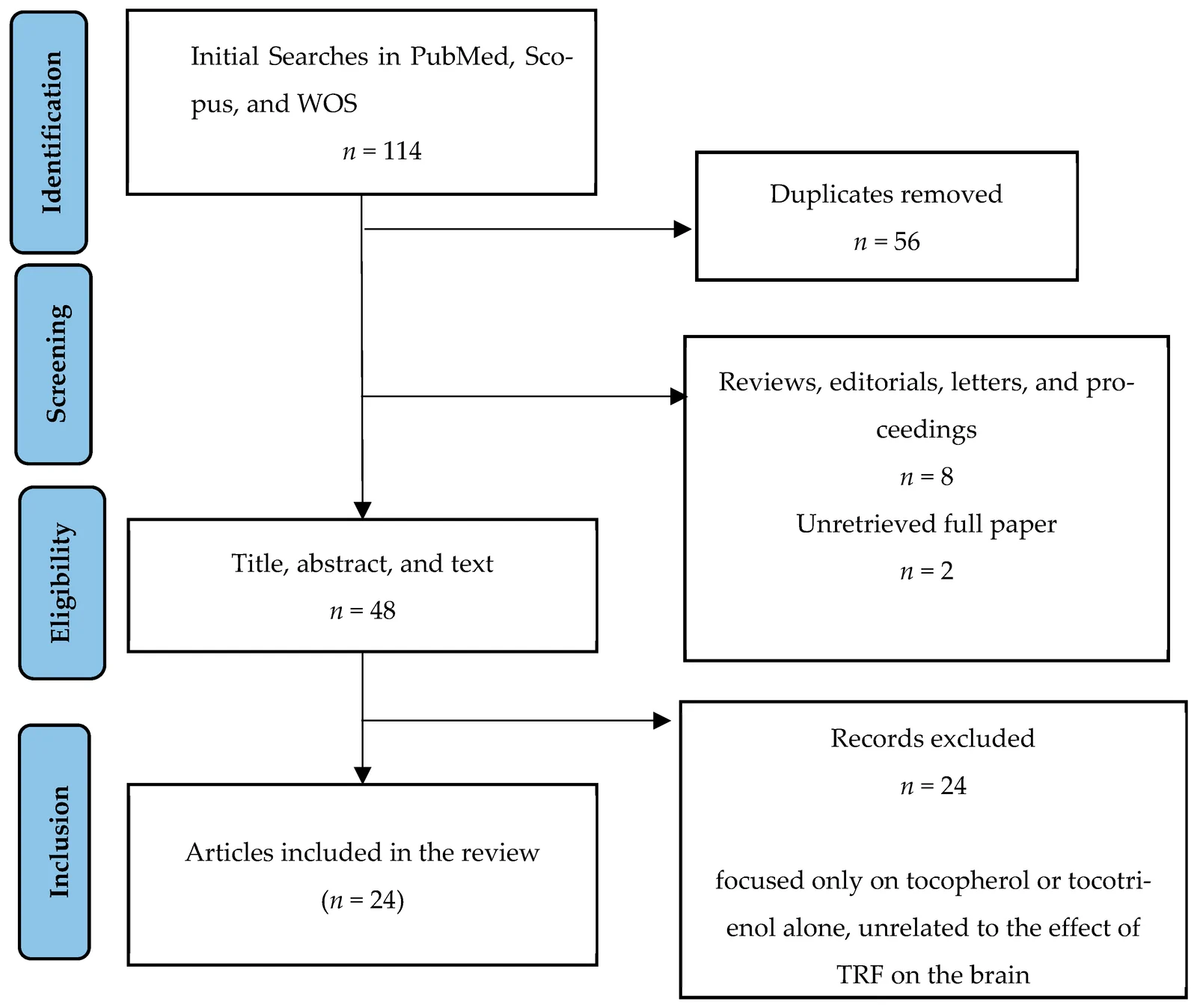
Articles screening using PRISMA flowchart as the study selection process.

Primary screening of the identified papers was conducted independently by the main investigators (E.Y. and M.L.N.) based on titles and abstracts. Any conflicts were resolved through discussion between the two reviewers, and no additional reviewer was required. The study selection process continued by applying predefined inclusion and exclusion criteria to all eligible papers. All authors participated in reviewing the final set of selected articles for eligibility. Figure 2 provides an overview of the study selection process. During this process, duplicate records, review articles, editorials, letters, conference proceedings, and papers that could not be retrieved due to inaccessible full texts were excluded.

### 4.4. Organizing and Recording All the Data

All the selected papers were organized based on their study characteristics. Specific study designs were also highlighted during data recording.

### 4.5. Summarizing the Key Points and Reporting the Results

Data from all eligible studies were extracted using a structured table format, systematically organizing information by study type, sample characteristics, and reported outcomes. The extracted outcomes specifically focused on the effects of TRF on brain cells, as defined by the parameters reported in each study.

## 5. Conclusions

TRF’s potential to confer neuroprotection presents a promising and cost-effective strategy in both healthy ageing and neurodegenerative conditions. We humbly believe that there exists a crucial link between mitochondrial dysfunction and microglial activity that underlies the protective abilities of TRF. However existing studies remain fragmented. There is yet a unified description of how TRF truly functions. Despite these glimpses of evidence, they are sufficient to lay the groundwork for future investigations.

## Data Availability

No data were generated from this study; all input was from the literature.

## Supplementary Materials

The following supporting information can be downloaded at: https://www.mdpi.com/article/10.3390/ijms26167691/s1.
