# Correction: Yunita et al. Scoping Review: The Role of Tocotrienol-Rich Fraction as a Potent Neuroprotective Agent. *Int. J. Mol. Sci.* 2025, *26*, 7691

**DOI:** 10.3390/ijms27114906

**Published:** 2026-05-29

**Authors:** Elvira Yunita, Muhammad Luqman Nasaruddin, Nur Zuliani Ramli, Mohamad Fairuz Yahaya, Hanafi Ahmad Damanhuri

**Affiliations:** 1 Department of Biochemistry, Faculty of Medicine, Universiti Kebangsaan Malaysia Medical Centre (UKM-MC), Kuala Lumpur 56000, Malaysia; elvirayunita@unib.ac.id (E.Y.); hanafi.damanhuri@ppukm.ukm.edu.my (H.A.D.); 2 Department of Biochemistry and Molecular Biology, Faculty of Medicine and Health Sciences, University of Bengkulu, Bengkulu 38371, Indonesia; 3 Department of Anatomy, Faculty of Medicine, Universiti Teknologi MARA, Sungai Buloh 47000, Selangor, Malaysia; zulianiramli@uitm.edu.my; 4 Department of Anatomy, Faculty of Medicine, Universiti Kebangsaan Malaysia Medical Centre (UKM-MC), Kuala Lumpur 56000, Malaysia; mfairuzy@ukm.edu.my

**Missing Citation**

In the original publication, Borsche M et al. was not cited. Previous paper (No. 66-Sliter, D.A. et al. was retracted by the publisher) [1]. So, the authors want to change the retracted paper [66] with a new reference. The citation has now been inserted in the Discussion Section, Fourth Paragraph and should read:

Likewise, in PD patients, a robust inflammatory response including IL-6, was observed following PRKN/PINK1, which promoted mtDNA release and mitochondrial damage [66].

**Original ref. 66**: Sliter, D.A.; Martinez, J.; Hao, L.; Chen, X.; Sun, N.; Fischer, T.D.; Burman, J.L.; Li, Y.; Zhang, Z.; Narendra, D.P.; et al. Parkin and PINK1 mitigate STING-induced inflammation. *Nature* **2018**, *561*, 258–262. https://doi.org/10.1038/s41586-018-0448-9.

**New ref. 66**: Borsche, M.; König, I.R.; Delcambre, S.; Petrucci, S.; Balck, A.; Brüggemann, N.; Zimprich, A.; Wasner, K.; Pereira, S.L.; Avenali, M.; et al. Mitochondrial damage-associated inflammation highlights biomarkers in PRKN/PINK1 parkinsonism. *Brain* **2020**, *143*, 3041–3051. https://doi.org/10.1093/brain/awaa246.

The authors state that the scientific conclusions are unaffected. This correction was approved by the Academic Editor. The original publication has also been updated.
