# Supplementary figures and images for: Scoping Review: The Role of Tocotrienol-Rich Fraction as a Potent Neuroprotective Agent
Source: Int J Mol Sci. 2025 Aug 8;26(16):7691. doi: 10.3390/ijms26167691 (PMC12386706; doi:10.3390/ijms26167691)

## Supplementary S1

### Result assessment of SYRCLE's RoB

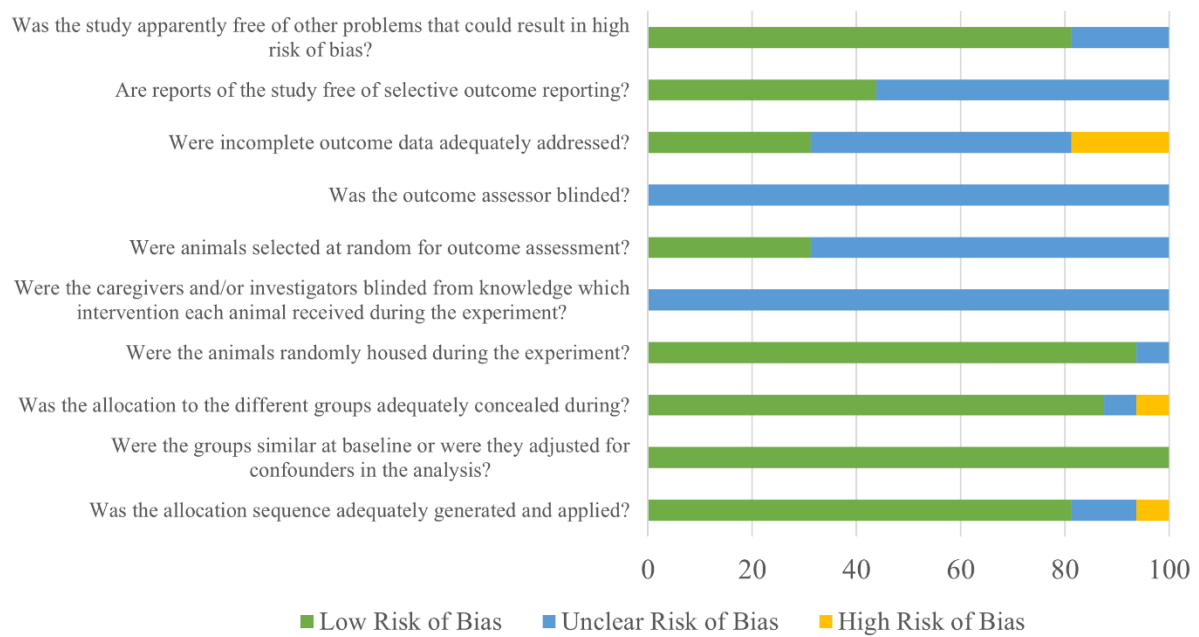

Supplement: Supplementary file 1 [file ijms-26-07691-s001.zip › Supplementary S1 (SYRCLE's RoB-Nita) 2.pdf]
